# Differential Modulation of Retinal Degeneration by *Ccl2* and *Cx3cr1* Chemokine Signalling

**DOI:** 10.1371/journal.pone.0035551

**Published:** 2012-04-24

**Authors:** Ulrich F. O. Luhmann, Clemens A. Lange, Scott Robbie, Peter M. G. Munro, Jill A. Cowing, Hannah E. J. Armer, Vy Luong, Livia S. Carvalho, Robert E. MacLaren, Frederick W. Fitzke, James W. B. Bainbridge, Robin R. Ali

**Affiliations:** 1 Department of Genetics, UCL Institute of Ophthalmology, London, United Kingdom; 2 Imaging Unit, UCL Institute of Ophthalmology, London, United Kingdom; 3 Department of Visual Science, UCL Institute of Ophthalmology, London, United Kingdom; 4 National Institutes of Health and Research Biomedical Research Center for Ophthalmology, Moorfields Eye Hospital, National Health Science Foundation Trust, UCL Institute of Ophthalmology, London, United Kingdom; Center of Ophtalmology, Germany

## Abstract

Microglia and macrophages are recruited to sites of retinal degeneration where local cytokines and chemokines determine protective or neurotoxic microglia responses. Defining the role of *Ccl2-Ccr2* and *Cx3cl1-Cx3cr1* signalling for retinal pathology is of particular interest because of its potential role in age-related macular degeneration (AMD). *Ccl2*, *Ccr2*, and *Cx3cr1* signalling defects impair macrophage trafficking, but have, in several conflicting studies, been reported to show different degrees of age-related retinal degeneration. *Ccl2/Cx3cr1* double knockout (*CCDKO*) mice show an early onset retinal degeneration and have been suggested as a model for AMD. In order to understand phenotypic discrepancies in different chemokine knockout lines and to study how defects in *Ccl2* and/or *Cx3cr1* signalling contribute to the described early onset retinal degeneration, we defined primary and secondary pathological events in *CCDKO* mice. To control for genetic background variability, we compared the original phenotype with that of single *Ccl2*, *Cx3cr1* and *Ccl2/Cx3cr1* double knockout mice obtained from backcrosses of *CCDKO* with *C57Bl/6* mice. We found that the primary pathological event in *CCDKO* mice develops in the inferior outer nuclear layer independently of light around postnatal day P14. RPE and vascular lesions develop secondarily with increasing penetrance with age and are clinically similar to retinal telangiectasia not to choroidal neovascularisation. Furthermore, we provide evidence that a third autosomal recessive gene causes the degeneration in *CCDKO* mice and in all affected re-derived lines and subsequently demonstrated co-segregation of the naturally occurring RD8 mutation in the *Crb1* gene. By comparing *CCDKO* mice with re-derived *CCl2^−/−^/Crb1^Rd8/RD8^, Cx3cr1^−/−^/Crb1^Rd8/RD8^* and *CCl2^−/−^/Cx3cr1^−/−^/Crb1^Rd8/RD8^* mice, we observed a differential modulation of the retinal phenotype by genetic background and both chemokine signalling pathways. These findings indicate that *CCDKO* mice are not a model of AMD, but a model for an inherited retinal degeneration that is differentially modulated by Ccl2-Ccr2 and Cx3cl1-Cx3cr1 chemokine signalling.

## Introduction

Mononuclear phagocytes (dendritic cells, macrophages, and microglia) are part of the myeloid cell lineage and are effector cells of the innate immune system. These cells all express CX3CR1 and are thought to be derived from different circulating monocyte populations [1]–[3]. In virtually all tissues, including the uveal tract of the eye, these cells form a network of resident dendritic cells and macrophages that is important for local immune surveillance and control of tissue homeostasis [3]–[5]. Microglia are the resident macrophages of the central nervous system including the retina and are located throughout the neural parenchyma (parenchymal microglia cells) and surrounding blood vessels (perivascular microglia cells) [6]–[9]. Resting microglia exhibit continuous, dynamic surveillance behaviour and maintain stable soma position [10], [11]. During acute and chronic degenerative changes in the retina, microglia as well as systemic macrophages become activated and are recruited to the site of tissue damage [12]. Such activation and recruitment of microglia and macrophages has been described in the retina during acute focal blue light damage [12], laser induced choroidal neovascularisation (CNV) [13], inherited retinal degenerations [9], [14], constant light exposure [15] as well as in other chronic inflammatory or para-inflammatory processes such as normal ageing [16], [17]. The local responses of microglia and macrophages are controlled by levels of cytokines and chemokines in the microenvironment which also define the heterogeneous activation state of these cells that can be pro-inflammatory (classical activation), cell or tissue protective (alternative activation) or immune suppressive [18]–[20]. In recent years, the two chemokine signalling pathways CCL2-CCR2 and CX3Cl1-CX3CR1, have become a major focus of ophthalmological research because of their proposed role in age-related macular degeneration (AMD), one of the most common causes for blindness in the elderly [21], [22]. CCL2-CCR2 signalling is one of the major pro-inflammatory pathways that controls transendothelial migration and recruitment of pro-inflammatory CCR2-expressing monocytes to sites of inflammation [23]–[25]. CX3CL1-CX3CR1 signalling controls the trafficking of patrolling monocytes [26] and regulates the communication of Cx3cr1 expressing microglia with neurons and glia in the nervous system to limit microglia-mediated neurotoxicity [23], [26]–[28]. In the retina, both pathways also control the dynamics and re-distribution of resting microglia during surveillance, activation/polarisation in response to acute injury or increased light and during ageing [15], [29], [30]. But it remains unresolved how these two signalling pathways interact in retinal degenerations and how defects in the pathways in the context of environmental (e.g. light) or endogenous (retinal degeneration) stress might alter the activation state of microglia or systemically derived macrophages and thereby might contribute to retinal pathology including photoreceptor and RPE degeneration during age-related retinal degeneration.

An important role of these two chemokine pathways in AMD has been supported by the identification of variants of the chemokine receptor gene *CX3CR1* as a genetic risk factor for AMD [29], [31]. However, this genetic association with AMD risk has not been reproduced in recent genome wide association and case-control studies [32]–[34]. Additional evidence for an important functional role of chemokine signalling during ageing and in AMD came from the observation of reduced expression of CX3CR1 in non classical monocytes and an increased serum level of CCL2 with age [35]. In addition, aqueous humor samples from AMD patients with exudative late stage AMD show also higher levels of CCL2 compared with controls [36]. Overall, this suggests that CX3CR1 and CCL2 chemokine signalling pathways not only play a role during normal ageing, but may be involved in the pathogenesis of AMD.

Several conflicting studies on single chemokine knockout mice for *Ccl2*, *Ccr2* or *Cx3c1* have reported that the abnormal trafficking and function of macrophages and microglia in these models is associated with a variable degree of age-related retinal and RPE degeneration [29], [37], [38]. A consistently described feature of all these chemokine knockout models are opaque yellow-white discrete spots in normal bright light fundus images. This phenotype has led to the misleading description of these mice as exhibiting drusen-like lesions and to the hypothesis that these mice represent models of general AMD pathology [29], [37]. The origin of the fundus lesions have recently been demonstrated to be bloated lipofuscin containing macrophages or microglia in the subretinal space that slowly accumulate with age [39], [40].

The age-related accumulation of subretinal macrophages in *Ccl2*, *Ccr2* and *Cx3cr1* knockout mice shows that chemokine signalling defects lead to dysfunctional macrophages in the retina. However, the contributory role of these dysfunctional macrophages to photoreceptor or RPE degeneration is not clear. Some groups have not observed significant age-related retinal and RPE degeneration or any spontaneous CNV in *Ccl2* knockout mice [40], while other groups have reported a variable penetrance of age-related photoreceptor and RPE degenerations as well as the spontaneous development of CNV in *Ccl2* and *Ccr2* single knockout mice [37], [38]. In *Cx3cr1* knockout mice, accumulation of subretinal macrophages/microglia is associated with a marked, progressive age-related retinal degeneration. This suggests that *Cx3cr1* signalling may play a more pronounced role for survival of photoreceptors and the RPE, while the role of *Ccl2* in this process remains unresolved [29], [40]. Overall these conflicting results suggest that additional factors contribute to the observed degenerative processes in chemokine knockout mice. These might involve environmental conditions such as housing, light, infection/pathogen burden and diet. Furthermore, *Ccl2/Cx3cr1* double knockout mice have been described as developing a more accelerated and severe, early onset retinal phenotype with high penetrance of rapid photoreceptor loss and RPE defects, which suggested that the combined knockout of both chemokine signalling pathways might act synergistically and may thus lead to an early onset retinal degeneration [41]. Since the original report, these mice have been studied by a number of laboratories and are considered to be a reasonable model for chemokine-mediated pathological processes contributing to AMD pathology. Several anti-inflammatory and anti-oxidative treatment approaches, e.g. with omega-3 fatty acids [42] as well as anti-VEGF [43] treatment have been reported to reduce lesion development in this mouse line.

In order to understand the reported phenotypic discrepancies in the different chemokine knockout mouse models and to study how defects in the two *Ccl2* and *Cx3cr1* signalling pathways may contribute to the development of the early onset retinal degeneration, we aimed to define primary and secondary pathological events during the age-related progression of the degeneration in *CCDKO* mice between 2 weeks and 22 months of age [41]. To control for genetic background variability, which might account for some of the reported phenotypic variability in retinal degenerations of chemokine knockout mice, we established single *Ccl2*, single *Cx3cr1* and double *Ccl2/Cx3cr1* knockout mice from the original *CCDKO* mouse line by backcrossing with *C57Bl/6* mice. We housed all these lines under same lighting conditions in the same room to control for environmental factors including pathogen burden and in addition raised some *CCDKO* mice in darkness from birth. Through these experiments, we identified the primary pathological event in *CCDKO* mice in the outer nuclear layer of the retina and established the *Crb1^RD8/RD8^* mutation as a third independent autosomal recessive locus as the cause for the early onset retinal degeneration which was not dependent on light. Furthermore, we observed differential modulatory effects of the genetic background, as well as of both chemokine signalling pathways on the manifestation of the early onset retinal degeneration and therefore show that *CCDKO* mice are not a model for AMD pathology. These finding also highlight a differential modulatory role of Ccl2-Ccr2 and Cx3cl1-Cx3cr1 chemokine signalling for retinal degeneration.

## Materials and Methods

### Animals and housing conditions

*Ccl2/Cx3cr1* double knockout mice (*CCDKO* mice) used in this study were derived from two breeding pairs (2 females and 2 males) that we obtained from the original line as described by Tuo et al. and were thankfully provided by Chi-Chao Chan and Jingsheng Tuo [41]. According to this publication the line was mainly of *C57Bl/6* background [41]. These animals were initially kept as a homozygous line under a normal 12-hour light-dark cycle. The mean luminescence during the light period at the level of bottom of the cage was 33±28 lx. Furthermore, the animals have access to cover (e.g. paper roll and excess of bedding) inside the cage which allows them to burrow. For dark housing (darkness luminescence <0.5 lx), pregnant female mice were transferred into a ventilated housing cabinet (Scantainer I-110, Scanbur, Denmark) which was further modified in house to reduce light exposure from the outside. Dark-housed animals were born in darkness and kept in the cabinet for 8 weeks before analysis. Animal husbandry was performed under red light (emission spectrum above 600 nm, red lamp, #02580, British Electrical Lamps Limited, UK).

As control animals we used age-matched *C57Bl/6J OLA Hsd* mice (Harlan UK Ltd., Blackthorn, UK) that were imported as young adult mice at 6–8 weeks of age and housed in the same animal rooms next to *CCDKO* mice. For backcrossing experiments, *CCDKO* mice from our original homozygous line and *C57Bl/6J Ola Hsd* mice were used as founder animals (F_0_). Obtained offspring (F_1_) that were heterozygous for both, the *Ccl2* and the *Cx3cr1* alleles were interbred to obtain the F_2_ generation. F_2_- animals were genotyped for both cytokine loci and interbred with each other to establish new lines for all combination of cytokine genotypes that include homozygous wildtype (*Ccl2^+/+^/Cx3cr1^+/+^*) mice, new single knockout mice for *Ccl2* (*Ccl2^−/−^/Cx3cr1^+/+^*) or *Cx3cr1* (*Ccl2^+/+^/Cx3cr1^−/−^*) as well as a new double knockout line for both *Ccl2* and *Cx3cr1* (*Ccl2^−/−^/Cx3cr1^−/−^*).

For *in vivo* procedures, mice were anesthetized by a single intraperitoneal (IP) injection of a mixture of medetomidine hydrochloride (1 mg/kg body weight; Domitor; Pfizer Animal Health, New York, NY), and ketamine (60 mg/kg body weight) in water. Whenever necessary, the pupils were dilated with 1 drop of 1% tropicamide. The animal experiments were performed in accordance with the ARVO Statement for the Use of Animals in Ophthalmic and Vision Research and under the UK Home Office project licence (PPL 70/1279).

### Genotyping

Genotyping PCR for the cytokine loci were performed using Go Tag Ready Green Mix (M712, Promega, Southampton, UK) using primer combinations distinguishing the wildtype and knockout alleles for *Ccl2* and *Cx3cr1* respectively (table 1) [44]. Genotyping of the RD8 (retinal degeneration 8) allele carrying a single base pair deletion (ΔC) in exon 9 of the *Crb1* gene was confirmed by PCR amplification using flanking primers and subsequent sequencing (table 1).

**Table 1:** PCR primer for genotyping of *Ccl2* and *Cx3cr1* alleles and for amplification of the *RD8* locus for sequencing.

Gene	Allele	Primer name	5′ – 3′ sequence
*Ccl2*	Wildtype	mmMCP1 ex1 for1	ctgtcatgcttctgggcctg
		mmMCP1 ex2 R1	cttgctggtgaatgagtagc
*Ccl2*	Knockout	MCP1 Neo Reverse	ctaggggaggagtagaaggtg
		mmMCP1 ex2 R1	cttgctggtgaatgagtagc
*Cx3cr1* [44]	Wildtype	Cx3cr1 WTP	ggcctgttatttgggcgacat
		Cx3cr1 ASP	tggggtgacgccactaagat
*Cx3cr1* [44]	Knockout	Cx3cr1 KOP	gaccgcttcctcgtgcttta
		Cx3cr1 ASP	tggggtgacgccactaagat
*Crb1*	RD8	mCrb1 F1	gcacaatagagattggaggc
		mCrb1 R1	tgtctacatccacctcacag

### Autofluorescence imaging and phenotyping of mice using scanning laser ophthalmoscopy

Autofluorescence imaging was performed using a HRA2 scanning laser ophthalmoscope with a 55° angle lense as described previously [40]. We used the autofluorescent mode of the HRA2 (Heidelberg engineering, Heidelberg, Germany) to scan the retina with a 488 nm laser that provides the excitation light to stimulate emission of autofluorescence from any possible fluorophores in the retina including e.g. lipofuscin in the RPE. The optic disc was positioned at the center of the image and the image focused either on the inner retinal vasculature, the inner retinal layer or the outer retina respectively. Projection images of 30 frames per fundus were taken and evaluated for the appearance of autofluorescence.

To phenotype offspring during backcrosses for retinal degeneration, we used fundus images focused on the inner retinal layer and evaluated the phenotype of the animals qualitatively using the red free (barrier filter at 500 nm) and the autofluorescent channels of the HRA2. To more quantitatively evaluate the severity of the phenotype, we subsequently counted the number of autofluorescent lesions per fundus image in the autofluorescent images for each animal after these had been processed in Adobe Photoshop CS 2 (Adobe Systems Incorporated, UK) using the auto level function. These counts include not only the typical disciform autofluorescent lesions from the inferior retina, but also include any autofluorescent signal from the whole fundus image including those from the superior retina.

### Optical coherence tomography (OCT) and fundus fluorescein angiography (FFA)

OCT imaging and fundus fluorescein angiography were performed during the same session using either the Spectralis™ HRA+OCT (Heidelberg engineering, Heidelberg, Germany) for OCT imaging or the HRA2 for fundus fluorescein angiography. First OCT images were obtained from the animals using OCT+IR or OCT+AF channels to correlate the retinal position with the obtained two-dimensional optical section obtained by the OCT. Subsequently, the same animals were injected intraperitoneal with 200 µl of 2% fluorescein in phosphate buffered saline (PBS) and fundus fluorescein angiography images were taken using the autofluorescent channel of the HRA2 equipped with an Argon laser at 488 nm wavelength. Obtained 2-dimensional OCT images and autofluorescent fundus images were exported and processed in Adobe Photoshop CS 2 (Adobe Systems Incorporated, San Jose, USA).

### Histopathology and pathology grading

Semithin histological morphometric analyses were performed as described previously [40]. Animals were sacrificed at the respective age and cardiac perfusion with 1%PFA was performed before the eyes were enucleated and fixed in 3% glutaraldehyde and 1% paraformaldehyde in 0.08 M sodium cacodylate-HCl (pH 7.4) for at least 30 hours at 4°C. The cornea and lens were removed and the eye cups oriented and post fixed in 1% aqueous osmium tetroxide for 2 hours, dehydrated by an ascending ethanol series (50%–100%) and propylene oxide, and infiltrated overnight with a 1∶1 mixture of propylene oxide. After 8 hours in full resin, the eyes were embedded in fresh resin and incubated overnight at 60°C. Semithin (0.7 µm) sections were cut in the inferior–superior axis passing through the optic nerve head with a microtome (Ultracut S; Leica, Wetzlar, Germany). Semithin sections were stained with a 1% mixture of toluidine blue-borax in 50% ethanol and images taken using bright field microscopy (Oberserver.Z1 Axio, Carl Zeiss Microimaging, Jena, Germany).

To evaluate the pathological changes in the retina and the RPE over time sections from age-matched *CCDKO* and *C57Bl/6* wildtype mice between 2 weeks and 22 months of age were assessed under bright field microscopy using a 100× objective and a 10× ocular lense. Singular pathological events in the retina (e.g. misplacement of nuclei from the outer nuclear layer toward the inner or outer retina or disorganized photoreceptor columns) and in the RPE (e.g. cell lysis, pyknosis, swelling, thinning, proliferation and thickening of RPE) were counted form the superior to the inferior end of the retina in each section. From these individual sums of damages per section we calculated the mean sum of outer nuclear and RPE damage from three sections per animal to obtain a representative measure of outer retinal or RPE damage score per animal similar to a scheme described by Hollyfield *et al*. and us [40], [45].

### Ultrastructural analysis using serial block-face scanning electron microscopy- (SB_SEM) with Gatan 3View

For 3-D analyses retinae fixed in 3% glutaraldehyde and 1% paraformaldehyde buffered to pH 7.4 with sodium cacodylate were rinsed in buffer, and then osmicated, en bloc stained with uranyl acetate and Waltons lead citrate using the method of West et al. [46] with two minor modifications. First, we reduced the osmium concentration to 1% and second, employed propylene oxide as a transition solvent between absolute alcohol and Durcupan ACM resin.

Regions of interest (ROI) were cut from resin blocks following normal surveying by LM and TEM of toluidine blue stained semithin sections and unstained ultrathin sections. The isolated ROI's were then superglued onto a Leica cryopin, re-trimmed to place the region of interest within a mesa of height ∼1 mm and side 0.5 mm and sputter coated with 5 nm gold palladium. Next, the specimen was locked into the specimen holder of the Gatan 3 View ultramicrotome mounted to the opened chamber door of a Zeiss Sigma variable pressure field emission scanning electron microscope and the diamond knife advanced until the full face of the block was being sectioned at 200 nm increments. At this point the microscope was evacuated and the block face imaged using Gatan's low voltage backscatter detector at 2–4 kV and chamber pressure of 10–30 Pa to suppress charging artifacts. Finally the pixel dwell time, magnification and chamber pressure were optimised to yield a focused ROI and the microtome programmed to automatically cut a maximum of 999×100 nm thick sections with intervening image acquisitions. In this way a stack of 999 images spanning an axial distance of 99.9 µms were digitally acquired at 4096×4096 pixel resolution in Digital Micrograph format. Three-dimensional reconstruction and labeling of the vascular lumen and Bruch's membrane was obtained using the Amira 5.3.3 software (Visage Imaging Inc., Berlin, Germany).

### Immunohistochemistry

Eyes for retinal and RPE/choroidal flat mounts were briefly fixed in 4% paraformaldehyde (PFA)/PBS before dissection and post-fixed again in 4% PFA/PBS for a total of 1 hour. After blocking with PBS/1% BSA (Sigma Aldrich, Steinheim, Germany)/5% nonspecific goat serum (AbD Serotec, Kidlington, UK) including 0.3% Triton X-100 for permeabilisation for 1 hour the flat mounts were incubated over night at 4°C with a 1∶500 dilution of anti Iba1 antibody (final concentration = 1 mg/ml, Code No. 019-19741, WAKO, Osaka, Japan) in blocking solution to label microglia and with primary TRITC-conjugated lectin at a 1∶10 dilution (final concentration = 0.1 mg/ml, L5264 Sigma Aldrich, Steinheim, Germany) to label endothelial cells. After washing with 3–4 times with PBS, 1∶500 dilution of goat anti-rabbit AlexaFluor 488 nm-conjugated secondary antibody (final concentration 4 µg/ml, #A11034; Invitrogen-Molecular Probes, Leiden, The Netherlands) was used to visualize the Iba1 antibody. After washing three times with PBS, retinal and RPE/choroidal flat mounts were mounted with fluorescence mounting medium containing Hoechst 33342 (Dako, Cambridgeshire,UK) and images were obtained using a confocal laser scanning microscope (Leica DM5500 Q, Leica Microsystems,Wetzlar, Germany). The obtained Z-stack images were processed for 3D data visualization using Imaris software (Bitplane, Zurich, Switzerland).

### Statistical analysis

Statistical analyses were performed using GraphPad Prism 5 for Windows (GraphPad Software Inc, La Jolla, USA).

## Results

### The early onset retinal degeneration in *CCDKO* mice starts in the inferior outer nuclear layer

**Figure 1:**
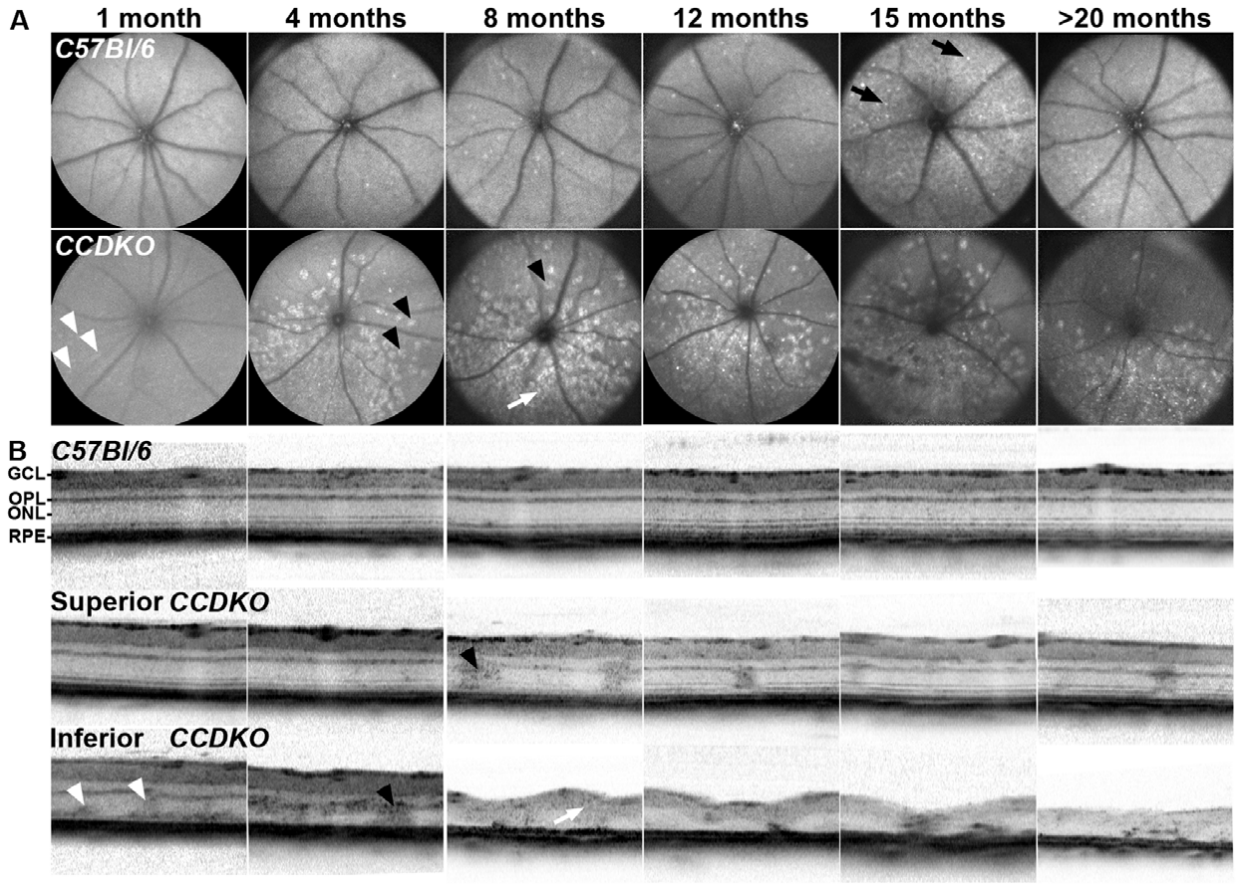
*CCDKO* mice show an early onset, focal inferior retinal degeneration starting in the outer retina which subsequently leads to a breakdown of retinal layering in the affected area with age. *In vivo* assessment of the retinal degeneration of *CCDKO* mice and age-matched *C57Bl/6* wildtype mice at 1,4,8,12,15 and older than 20 months of age using autofluorescence scanning laser ophthalmoscopy (A) and optical coherence tomography (B). Note the weak autofluorescent fundus lesions in *CCDKO* mice visible as early as 1 month (A) which correlate with the observation of an abnormal signal in the inferior OCT image at the same age (B) and suggests an early pathological event in the outer retina as the basis of the degeneration in this model. Corresponding signals in AF-SLO and OCT images are labelled identically. White arrowheads: small early inner retinal autofluorescent lesion, black arrowheads: discrete disciform autofluorescent lesion, white arrow: “confluent” autofluorescent lesion in AF-SLO represents loss of retinal layering and severe retinal degeneration, black arrow: discrete autofluorescent spot-like lesion. GCL: ganglion cell layer, OPL: outer plexiform layer, ONL: outer nuclear layer, RPE: retinal pigment epithelium.

To identify the primary site of pathology and define secondary consequences during disease progression, we evaluated the spatial and temporal distribution of degenerative events in *CCDKO* line [41] in comparison to age-matched *C57Bl/6* mice between 1 month and 22 months of age. Autofluorescent fundus imaging by scanning laser ophthalmoscopy (AF-SLO) demonstrated faint abnormal autofluorescent fundus lesion at 1 months of age (figure 1A, white arrowheads) that evolved to medium sized, discrete disciform hyperfluorescent lesions at 4 months of age in the inferior retina of *CCDKO* mice (figure 1A, black arrowheads). *C57Bl/6* mice of any age between 1 month and 20 months did not exhibit similar lesions, but showed small, discrete autofluorescent fundus spots (figure 1A, upper row, black arrows), which we have previously described as a signal originating subretinal macrophages containing lipofuscin in aged mice [40]. The number of the inferior disciform lesions, which are more anterior located within the retina than subretinal macrophages (data not shown), increase with age and become confluent at later time points (figure 1A, lower row, white arrow). This indicates that *CCDKO* mice show a progressive early onset, inferior retinal degeneration that remains constricted to a focal area of the inferior retina even up to very late stages.

To further elucidate the location of autofluorescent fundus lesions within the retina, we performed optical coherence tomography (OCT, figure 1 B) in parallel to AF-SLO imaging (figure 1 A) in *CCDKO* and age-matched *C57Bl/6* controls between 1 and 22 months of age. *CCDKO* mice demonstrated abnormal OCT signals in the outer retina in particular within the outer nuclear (ONL) and the outer plexiform layer (OPL) which corresponded spatially to the disciform autofluorescent fundus lesions in the inferior retina (figure 1 B, white and black arrowheads). In line with the SLO data, these OCT changes in the inferior retina increase with age and lead to the disruption of retinal layering as well as retinal thinning and degeneration at 8 month of age and older (white arrow in figure 1A and figure 1B). *C57Bl/6* control mice did not show any abnormal OCT signals at any of the studied ages (1→20 months, upper row, Figure 1B).

Compared to this prominent, age-related progressive retinal degeneration observed in the inferior retina, the superior retina remained largely unaffected, despite some abnormal OCT signals obtained from areas close to the optic disc (e.g. figure 1B, 8 months, middle row, black arrowhead). These also correlated with some individual autofluorescent lesions observed in the AF-SLO fundus images in the superior retina (e.g. figure 1A, 8 months, lower row, black arrowhead). Taken together, these data indicate that the retinal degeneration in *CCDKO* mice starts primarily within the inferior outer retina and progresses in an age-dependent manner to a focal loss of all retinal layers in the inferior retina.

### The inferior localization of the retinal degeneration in *CCDKO* mice is not dependent on light

**Figure 2:**
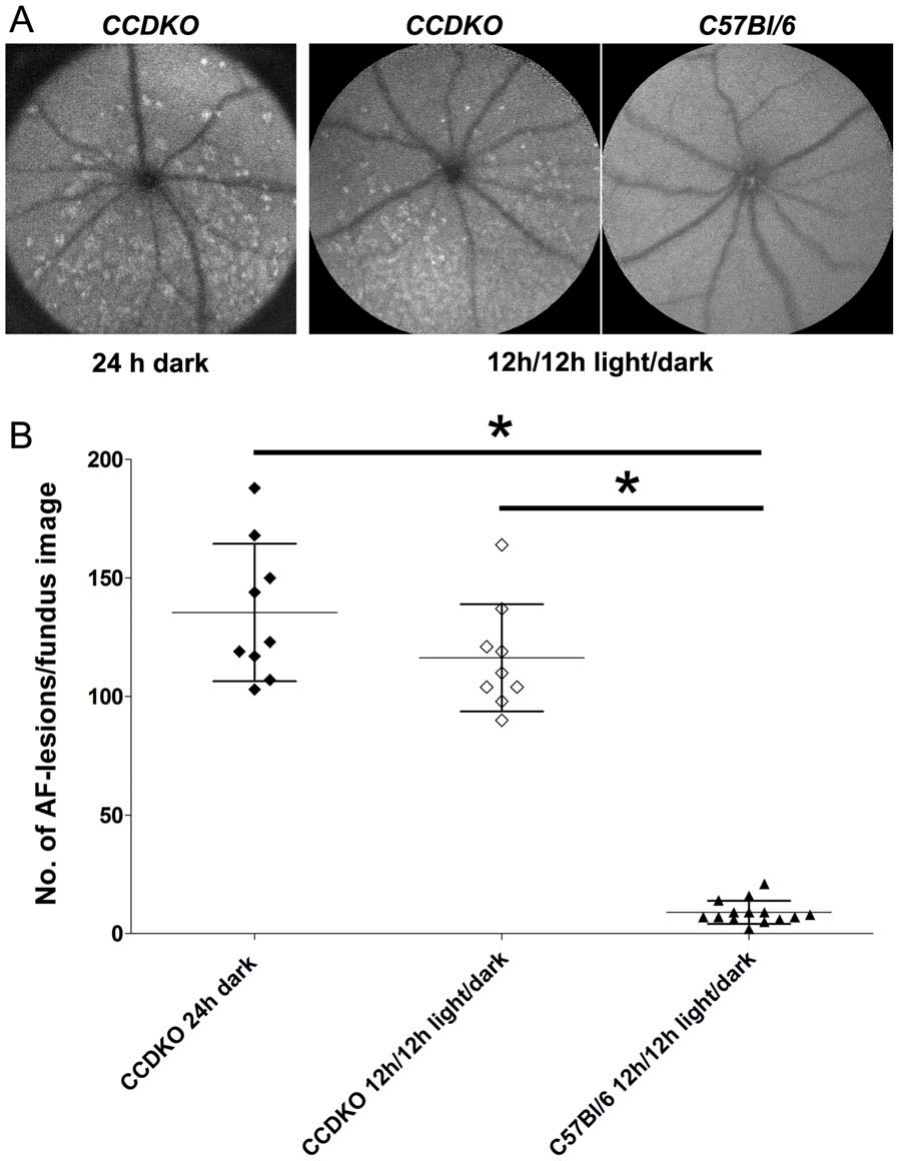
The inferior retinal degeneration in *CCDKO* mice is not dependent on light. (A) Comparison of autofluorescent SLO fundus images of *CCDKO* mice at 8 weeks of age that were raised from birth either in complete darkness (24 h dark, luminescence <0.5 lx) or in normal 12 h/12 h light/dark cycle (light 12 h/12 h, luminescence = 33±28 lx). As a comparison an AF-SLO fundus image from an aged-matched *C57Bl/6* wildtype mouse raised under normal 12 h/12 h light/dark cycle is shown. (B) Quantitative assessment of the number of autofluorescent lesions/per fundus image for the three animal groups. The number of autofluorescent lesions in *CCDKO* (24 h dark, n = 9) and *CCDKO* (12 h/12 h light/dark, n = 9) was not significantly different, but was significantly higher compared to that in *C57Bl/6* mice (12 h/12 h light/dark, n = 14). *: One-way-ANOVA (p<0.0001), Tukey's posthoc test for multiple comparison (p<0.05). This suggests that light is not necessary for the manifestation of the early onset inferior retinal degeneration in *CCDKO* mice and that ambient light (33+−28 lx) does not augment the number of inferior retinal autofluorescent lesions in *CCDKO* mice within the first 8 weeks of their life.

The inferior localization of the retinal degeneration observed in *CCDKO* mice suggested a possible role of light as an initiating or contributing factor for the development of the retinal degeneration. To evaluate, whether light is a necessary factor for this inferior retinal degeneration, we used autofluorescent SLO fundus imaging and compared the phenotype of *CCDKO* mice at 8 weeks of age which were raised from birth either in normal 12 h/12 h light/dark cycle conditions (33±28 lx during the light period) or in complete darkness (luminescence <0.5 lx). As an additional control group we included wildtype *C57Bl/6* mice raised under 12 h/12 h light/dark cycle conditions. Both groups of *CCDKO* mice showed a very similar inferior localisation of autofluorescent lesions at 8 weeks of age (Figure 2A), while control wildtype animals do not show any of the typical autofluorescent lesions in the inferior retina. This suggests that the manifestation of the inferior retinal degeneration in *CCDKO* mice is independent of light. To further evaluate, whether darkness might have attenuated the inferior retinal phenotype in dark raised *CCDKO* mice, we determined the number of autofluorescent lesions per fundus image in all three groups (figure 2B). We did not detect a significant difference in the number of autofluorescent lesions per fundus image between both groups of *CCDKO* mice, but observed a significantly higher number of autofluorescent lesions in *CCDKO* mice in general compared to *C57Bl/6* controls (Figure 2B, One-way ANOVA p<0.001, Tukey's Multiple comparison posthoc test p<0.05). These data further support that the manifestation of the inferior retinal degeneration in *CCDKO* mice is not dependent on light and that ambient light levels (luminescence = 33±28 lx) do not modulate the retinal phenotype in *CCDKO* mice significantly during the first 8 weeks of life.

### Autofluorescent retinal fundus lesions in the inferior retina are located in outer retinal layers and originate from columns of degenerating photoreceptor nuclei surrounded by recruited microglia

**Figure 3:**
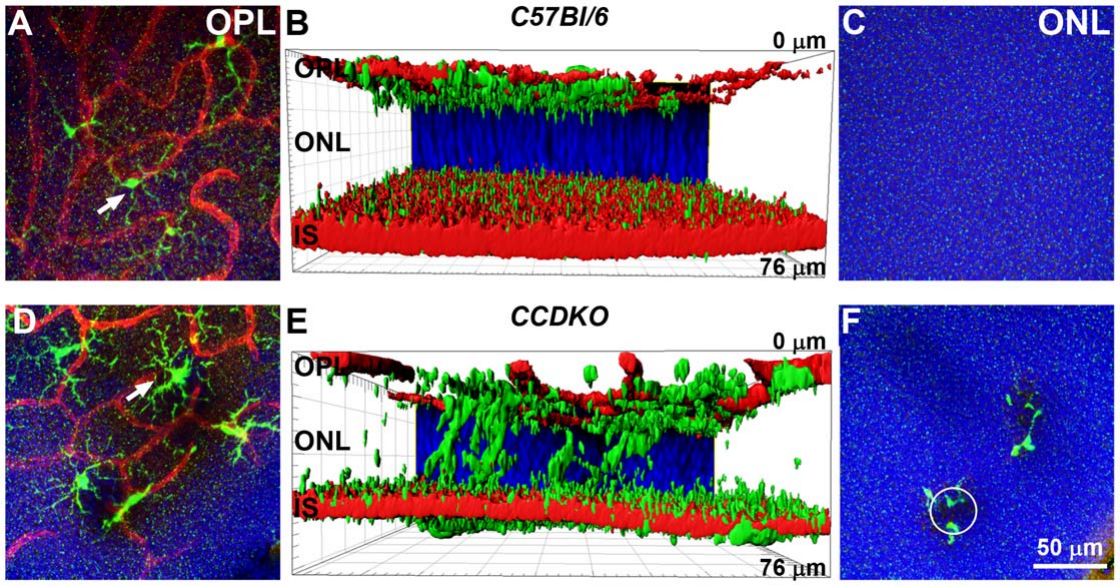
Microglia are recruited to the primary retinal lesions in the outer retina of *CCDKO* mice. Confocal maximum projection images of a subset of images at different depth of the z-stack (A,C,D,F) and images of 3D-reconstructions (IMARIS) of corresponding complete z-stacks (B,E). Z-stack images were taken from the inferior area of retinal flat mount preparations from *CCDKO* and *C57Bl/6* mice at 2 months of age. The samples were labeled with Tritc-lectinB4 (vascular marker, red), Iba1 (microglia marker, green) and DAPI as a nuclear counter stain (blue). OPL: outer plexiform layer, ONL: outer nuclear layer, IS: inner segments. (A+D) At the level of OPL ramified microglia (green) are located between vessels of the deep capillary plexus (red). Microglia of *CCDKO* mice (white arrows, D) show a slightly bigger cell bodies than those in *C57Bl/6* mice (A). Microglia were also observed in the outer retina of *CCDKO* mice in the 3D Imaris reconstruction (E) and in confocal z-projections from the outer retina (F) while they were not observed in the outer retina of *C57Bl/6* mice (B, C). microglia in the outer retina of *CCDKO* mice surround circular lesions (F, white circle) that may correspond well with the disciform shape of autofluorescent lesion observed in AF-SLO fundus images.

To further characterise the nature of the autofluorescent lesions in *CCDKO* mice we compared the localisation of AF-SLO and OCT lesions (Figure 1A and 1B) with that of lesions in retinal flat mount preparations stained for blood vessel and microglia marker, since microglia cells are known to be recruited to sites of retinal degenerations and can be altered in chemokine knockout mice (Figure 3 A, B, C, D, E, F, see also supplementary Movie S1 *C57Bl6* and supplementary Movie S2 *CCDKO*).

At the level of the deep capillary plexus in the outer plexiform layer (OPL, figure 3A and 3D), *CCDKO* mice demonstrated relatively normal ramified microglia which showed a slightly more swollen cell body than microglia in *C57Bl/6* mice at the same retinal layer (white arrows in figure 3D (*CCDKO*) versus 3A (*C57Bl/6*)). In the outer retina, however, *CCDKO* mice revealed microglia cells that have migrated into the outer nuclear layer (figure 3E, 3F, supplementary Movie S1 *CCDKO*) and are positioned around circular columns of photoreceptor nuclei dropping out of the ONL (white circle, figure 3F). In contrast, in *C57Bl/6* mice microglia are not present in the outer nuclear layer (ONL) or the inner segment (IS) area (figure 3B, C, supplementary Movie S1 *C57Bl6*). The observed degenerating columns of photoreceptor cells in *CCDKO* mice together with the surrounding recruited microglia correspond in location, size and configuration well with the disciform shape of the autofluorescent fundus lesions observed in AF-SLO images (figure 1A, black arrowheads) as well as with the OCT signals observed in the outer retina of *CCDKO* mice (figure 1B, white and black arrowheads). This data suggests that microglia in *CCDKO* mice are recruited to the site of primary retinal lesions in the outer retina of *CCDKO* mice and that this process and the lesion development is reflected by these autofluorescent fundus lesions observed *in vivo* by AF-SLO imaging.

### A localized drop-out of ONL nuclei suggests a primary retinal event to initiate the progressive inferior retinal degeneration in *CCDKO* mice which leads to late secondary RPE damage

**Figure 4:**
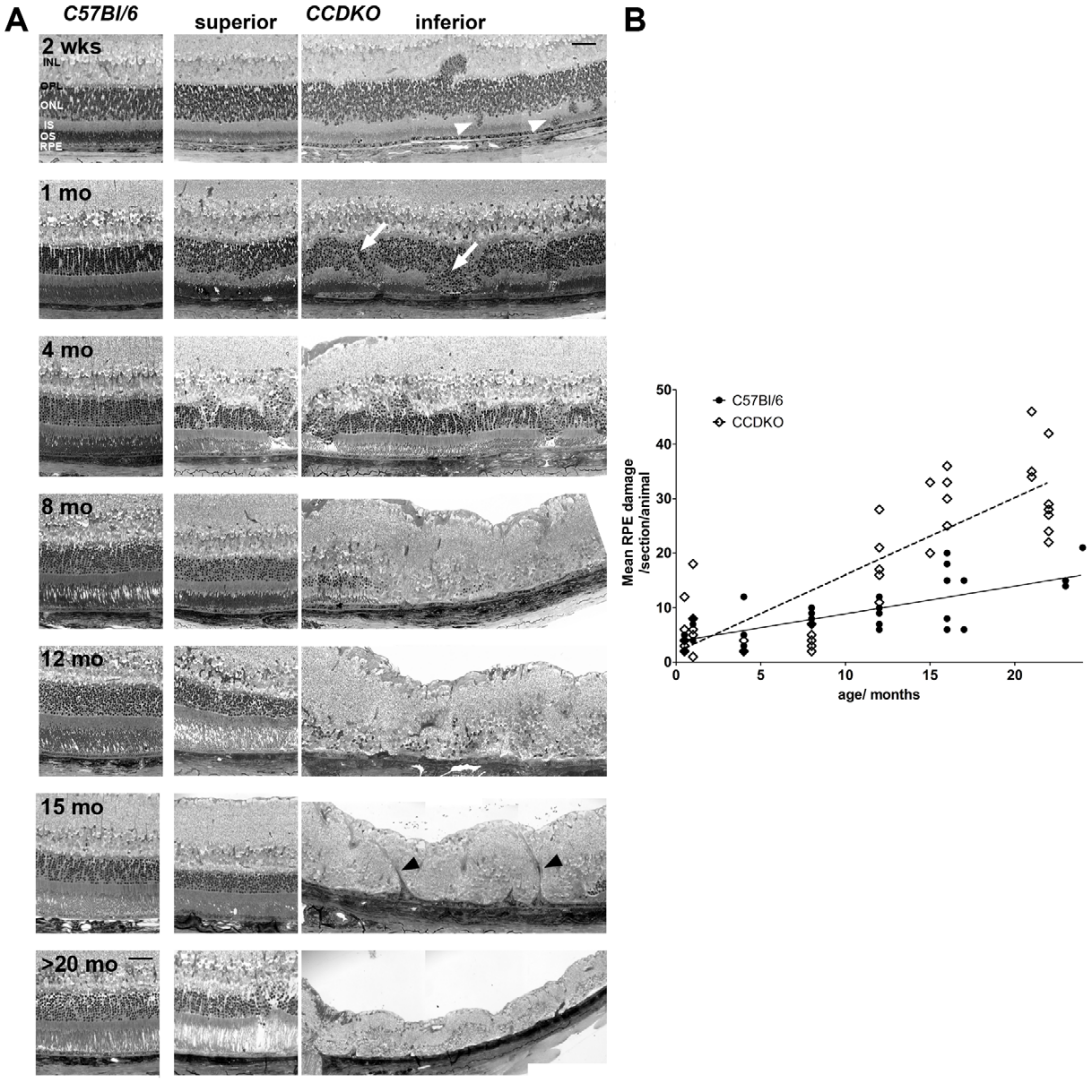
The primary degenerative event in the course of the degeneration is located in the outer nuclear layer of *CCDKO* mice and leads to the secondary development of RPE damage. (A) Superior-inferior oriented sagittal semithin histology of the outer retina of *CCDKO* mice and age-matched *C57Bl/6* wildtype mice at the level of the optic disc between 2 weeks and over 20 months of age. A localized drop-out of nuclei from the ONL (white arrowheads) as early as 2 weeks of age in *CCDKO* mice suggests a primary pathological event in the retina that initiates the progressive inferior retinal degeneration in this model finally leading to the complete loss of retinal layers from 8 months of age onwards. The RPE underneath these lesions is secondarily affected. White arrows: drop out of photoreceptor columns, black arrowheads: descending retinal vessels, INL: inner nuclear layer, OPL: outer plexiform layer, ONL: outer nuclear layer, IS: inner segments, OS: outer segments, RPE: retinal pigment epithelium. (B) Quantitative morphometry of RPE damage on the same sections. We observed an normal age-related increase of RPE damage in wildtype mice (*C57Bl/6*: Pearson r^2^ = 0.5562, p<0.0001, N = 37) and observed that the age-related increase of RPE damage in *CDDKO* mice (*CCDKO*: Pearson r^2^ = 0.7115, p<0.0001; N = 43) was significantly higher compared to *C57Bl/6* mice (linear regression analysis, p<0.0001),which was significantly more pronounced in *CCDKO* mice (Pearson r^2^ = 0.465,p = 0.0007; slope difference: p = 0.01). Nevertheless the RPE damage becomes only significantly higher during late stages of the degeneration suggesting a secondary involvement of the RPE during the degenerative process. Scale bars: 25 µm.

**Figure 5:**
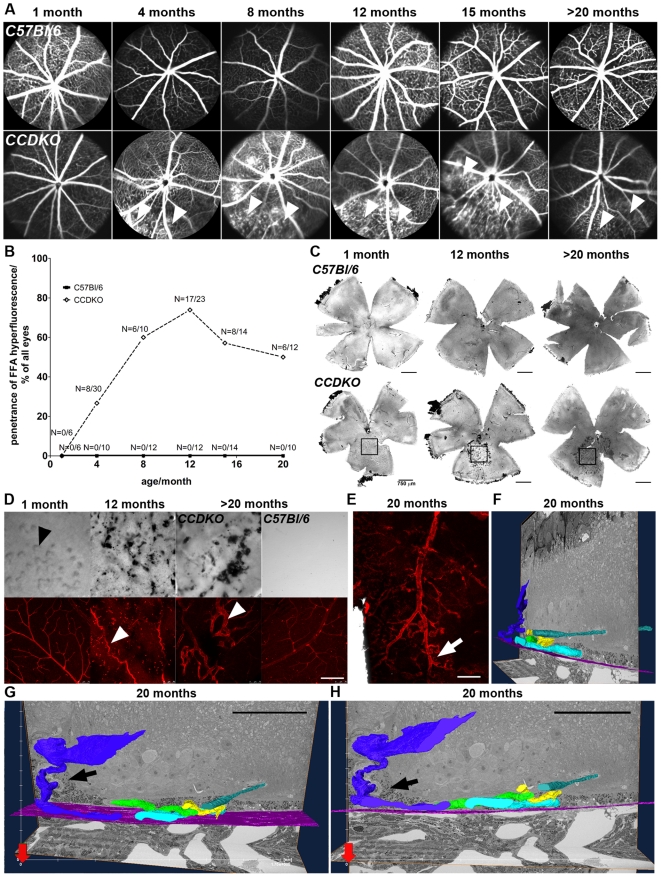
Vascular lesions in *CCDKO* mice develop secondarily to the retinal degeneration and show similarities to retinal telangiectasia rather than choroidal neovascularisation. (A) Late phase fluorescein angiography images of *CCDKO* mice and age-matched wildtype mice between 1 month and over 20 months of age reveals a late phase (∼7 min post i.p. injection) hyperfluorescence of fluorescein (white arrowheads) secondary to the focal early onset, inferior retinal degeneration. (B) Quantification of the penetrance (%) of late stage hyperfluorescence in *CCDKO* mice at different ages in comparison to *C57Bl/6* mice. The number of animals (N) analysed per age group and genotype are shown in the graph (N = eyes with late FFA hyperfluorescence/total number of imaged eyes). (C) DIC bright field images of retinal flat mount preparations of *CCDKO* and age-matched wildtype controls at 1 months, 12 months and 20 months, scale bar: 750 µm. (D) Magnified DIC bright field images and corresponding immunohistochemistry staining for the vascular marker isolectin B4 (TRITC-lectin-B4, red) taken from the affected inferior degenerate areas (black squares in (C)).At 1 month, early separate grey spots (5D, black arrowhead) were observed inferior to the optic disc, which later grow bigger, become confluent and involve pigmented cell attached to the focal inferior retinal lesion. Lectin-positive vessels within the area of degeneration show vascular anomalies secondary to the retinal degeneration, including vascular tortuosity (5D, white arrowhead, at 12 months) and dilation of retinal capillaries (5D, white arrowhead, at 20 months).Scale 150 µm. (E) Overlay of a bright field and immunohistochemistry image taken from the apical side of a lectin-stained RPE/choroidal flat mount from a *CCDKO* mouse at 20 months of age. The retinal vasculature within the degenerate area (white arrow) was closely attached to the underlying apical side of the RPE. Scale: 100 µm. (F–H): Different views of a three dimensional reconstruction from a tissue block from the centre of an inferior retinal lesion of a *CCDKO* mouse at 20 months of age using serial block-face scanning electron microscopy with a Gatan 3-View system. Connected vascular lumens are labeled by an identical color, while the location of Bruch's membrane in each ultrathin section is outlined by a pink line. Retinal vessels collapse onto the RPE and induce a migratory response of RPE cells to grow along these vessels into the retina (black arrows). Retinal vessels grow underneath the RPE and become positioned in close vicinity to Bruch's membrane within a RPE/vascular complex, but they never have been observed to grow through Bruch's membrane (Representative 3D reconstruction images from a tissue block with the dimensions; x-axis 176.42 µm, y-axis 176.42 µm, z-axis: 39.9 µm, Scale (G&H): 50 µm.

To evaluate the histopathology and the progression of the primary lesion and define secondary events during the retinal degeneration of *CCDKO* mice, we examined superior-inferiorly-oriented sagittal semithin sections for all age groups of *CCDKO* mice and control *C57Bl/6* mice. *CCDKO* mice demonstrate an early mislocalisation of photoreceptor nuclei in the inferior outer nuclear layer at two weeks of age indicating a very early onset of retinal changes in the inferior outer retina (Figure 4 A, white arrowheads). This phenotype becomes more prominent with age and whole columns of ONL nuclei drop out of the outer nuclear layer at 1 to 4 months of age (Figure 4A, white arrows). This leads subsequently to a prominent focal loss of all retinal layers in the inferior retina as well as to retinal thinning and scaring at ages between 8 and 12 months. Due to the retinal thinning, descending retinal vessels (figure 4A, black arrowheads) come in contact with the RPE and form RPE/vascular lesions that might contribute to retinal tractions and scaring. Within these lesions retinal vessels break through the RPE but do not to link up to choroidal vessels (see also figure 5 F, G, H and text below).

*CCDKO* mice have been reported to exhibit severe RPE damage [47] and there are conflicting reports for the degree of RPE changes with age in other *Ccl2* and *CCR2* single knockout strains [38], [40]. Therefore we aimed to elucidate the temporal onset of RPE damage in *CCDKO* mice between 1 and 22 months of age in order to understand, whether RPE damage is a primary or secondary event during the course of the degeneration. By using a previously described quantitative morphometry approach for grading RPE damage in mice [40], [45], [48], we found that the RPE damage in both, *CCDKO* and *C57Bl/6* control mice increased significantly with age (figure 4B, *C57Bl/6*: Pearson correlation r^2^ = 0.5562, p<0.0001, N = 37; *CCDKO* mice: Pearson r^2^ = 0.7115, p<0.0001; N = 43). The age-dependent increase in RPE damage in *CCDKO* mice was significantly higher compared to *C57Bl/6* mice indicating a disease related increase of RPE damage in *CCDKO* mice (figure 4B, linear regression analysis, slopes of the two curves are significantly different, p<0.0001). To evaluate the onset of increased RPE damage in *CCDKO* mice we compared the RPE damage score for each age group independently. While the RPE damage of *CCDKO* mice compared to age-matched *C57Bl/6* mice between 1 month and 8 months was not significantly increased, from 12 months onwards the RPE damage was significantly higher in *CCDKO* (figure 4B, One way ANAVO p<0.0001 with Bonferroni multiple comparison posthoc test p<0.05 was not significant at 0.5 month (m): N = 5/6; at 1 m: N = 3/5; at 4 m: N = 6/4 and at 8 m: N = 6/6, while it was significant at 12 m: N = 6/6; at 16 m: N = 7/7; and at >20 m: N = 4/9). This indicates that the RPE is affected secondarily and late during disease progression in *CCDKO* mice. In addition RPE damage occurs in particular in the area of focal retinal degeneration supporting that a primary retinal pathology leads to RPE damage in *CCDKO* mice (figure 4A, see also figure 5 C, D, E, F, G, H).

### The focal inferior retinal degeneration in *CCDKO* mice leads to the secondary development of vascular malformation similar to retinal telangiectasia

Previous work has suggested, that chemokines and in particular *Ccl2* and *Ccr2* [37], [40], [49] as well as *Cx3cr1* signalling [29] play an important role during the development of choroidal neovascularisation. Furthermore it has been reported that about 10–15% of *CCDKO* mice older than 3 months develop choroidal neovascularisation based on histological analysis and the finding that vessels breach the RPE [41]. To further understand the nature and the development of the vascular phenotype of *CCDKO* mice in relation to the progression of the retinal degeneration, we performed *in vivo* fluorescein angiography in *CCDKO* mice and age-matched *C57Bl/6* mice between 1 month and 20 months of age (figure 5A). Furthermore, we stained the vasculature in retinal and choroidal flat mount preparation with TRITC-conjugated isolectin B4 and analysed the vascular phenotype in *CCDKO* mice at different ages (figure 5C, D, E). Fluorescein angiography was performed on the same animals and in the same experimental session as the AF-SLO imaging (figure 1A) and OCT (figure 1B), which enabled us to directly compare the location of autofluorescent lesions and vascular changes in these animals. During the early retinal degenerative events at 1 month of age, *CCDKO* mice did not reveal any vascular changes in compared with control mice (figure 5 A, 1 month). In fluorescein angiography, *CCDKO* mice older than 1 month of age showed prominent areas of fundus hyperfluorescence (figure 5A, white arrowheads), but only low-grade evasation of fluorescein in the inferior retina during the late phase of the angiography (∼7 min, figure 5A, white arrowheads). These areas of fundus hyperfluorescence were observed in the same areas as the inferior retinal degeneration indicated in corresponding AF-SLO and OCT images by autofluorescent lesions (figure 1A, 1B). During later stages of disease, *CCDKO* mice demonstrate an increasing penetrance of the late-phase hyperfluorescence with age and about 50–74% of *CCDKO* mice exhibit such hyperfluorescent areas at ages between 12–20 months (figure 5B).

Retinal flat mounts from *CCDKO* and age-matched wildtype controls at 1 months, 12 months and 20 months imaged by interference contrast (DIC) bright field microscopy revealed grey spots in the inferior retina of 1 months old *CCDKO* animals (figure 5C, black square and 5D, black arrowheads) which correspond well to the location of autofluorescent lesions (figure 1A, white arrowheads) and where absent in *C57Bl/6* wildtype controls (figure 5C, upper row left and 5D, upper row right). At later time points during disease progression, at ages of 12 months and 20 months, *CCDKO* mice show an increasing number of pigmented cells located within the area of retinal degeneration (figure 5C, black squares correspond to the areas from which images in figure 5 D were taken). These pigmented cells are pigmented RPE cells that are attached to the retina from the subretinal side and have infiltrated along the retinal vasculature that came in touch with the RPE (figure 5G, arrow). Only within areas of pronounced inferior retinal degeneration at 12 month and >20 months, we observed retinal vascular anomalies including vessel tortuosity (figure 5D, white arrowhead e.g. at 12 months), dilated vessels (figure 5D, white arrowhead, e.g. at >20 months) and retinal vessels that were closely attached to the RPE, e.g. shown on an apical image of an isolectin B4 stained RPE/choroidal flat mount preparation to which the retinal vasculature within the degenerate areas was attached (figure 5E, white arrow). Three-dimensional reconstruction of tissue blocks within the degenerate area of aged *CCDKO* mice by serial block-face scanning electron microscopy (figure 5F–H) demonstrate that retinal vessels within the degenerate area are severely abnormal (figure 5F) and are directly connected to vessels that are positioned adjacent to Bruch's membrane underneath the RPE (figure F, G). RPE cells show a migratory response along the descending retinal vessels that leads to an RPE/Vascular complex in which RPE cells surround retinal vessels within the retina (figure 5G, black arrow). These vessel correspond in location well with the directly attached retinal vasculature observed apically on the RPE/choroidal flat mount (figure 5E, white arrow) and the localisation of RPE cells within the retinal flat mount preparations in the area of degeneration (figure 5C&D). Translocated retinal vessels that are closely positioned adjacent to Bruch's membrane do grow along Bruch's membrane within the lesion area, but have not been observed, neither on the semithin sections nor in three different ultrastructural reconstructions, to break through Bruch's membrane (figure 5H) suggesting that Bruch's membrane remains intact in the *CCDKO* mouse model even underneath very late vascular lesions. These clinical, immunohistological and ultrastructural data suggest that *CCDKO* mice develop vascular lesions secondarily to the observed retinal degeneration. The vascular anomalies are of retinal origin and do not connect to the choroidal vasculature and rather share similarities with retinal telangiectasia than with clinical features of choroidal neovascularisation.

### The early onset, inferior retinal degeneration does segregate independently of either of the chemokine knockout alleles (*Ccl2* or *Cx3cr1*) in an autosomal, recessive Mendelian trait

**Figure 6:**
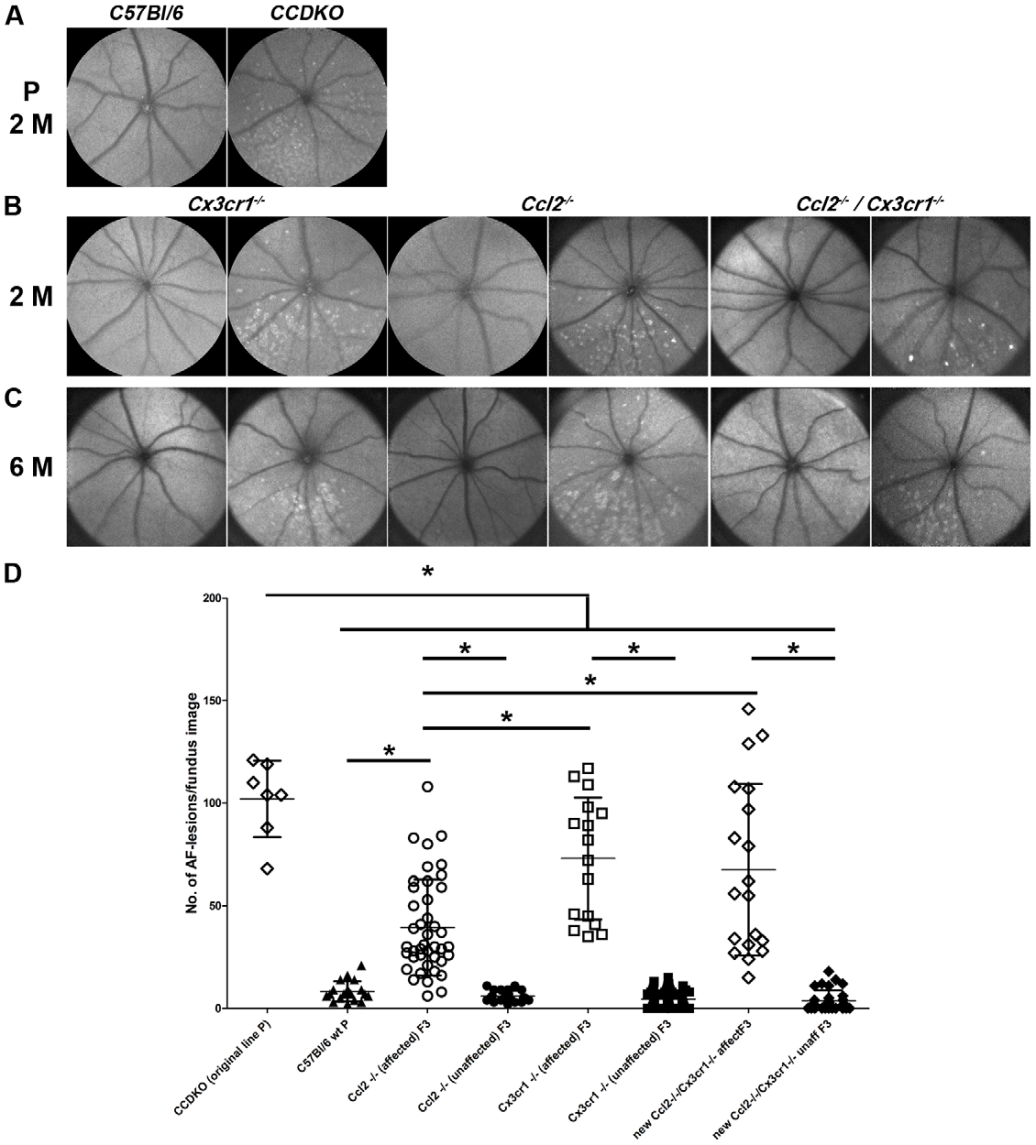
*Ccl2* and *Cx3cr1* as well as the genetic *C57Bl/6* background differentially modulate the autosomal recessive early onset, inferior retinal degeneration caused by the RD8 mutation in exon 9 of the *Crb1* gene. *In vivo* phenotyping by autofluorescent fundus imaging of the two parental (P) mouse lines (*CCDKO* and *C57Bl/6*) (A) as well as in the newly established chemokine knockout mouse lines (*Ccl2^−/−^, Cx3cr1^−/−^* and *Ccl2^−/−^/Cx3cr1^−/−^*) at the age of 2 months (2 M, B) and 6 months (6 M, C). Autofluorescent fundus lesions were observed independently from the chemokine genotypes at the age of 2 months and 6 months suggesting that an independent genomic locus is responsible for the early onset, inferior retinal degeneration. (D) Quantification of the number of autofluorescent lesions per fundus image for all parental, affected and unaffected chemokine knockout mouse lines indicated significant differences in the number of autofluorescent fundus lesions between the groups. The individual number of autofluorescent lesions per fundus and animal is shown together with the mean ± standard deviation. *: (One-way-ANOVA p<0.0001, Tukey posthoc test for multiple comparison (p<0.05)). This data indicate an attenuating influence of increasing *C57Bl/6* genetic background in all offspring obtained from the F2 generation relative to the parental *CCDKO* mouse line and thus were labelled as F3 generation. A differential modulatory effect of both chemokine signalling pathways on the severity of the retinal degeneration at 8 weeks of age was also observed by comparing the three affected lines within the F3 generation. N (*CCDKO* original line P) = 7, N(*C57Bl/6 wt* P) = 19, N(*Ccl2−/−* affected) = 42, N(*Ccl2−/−* unaffected F3) = 23, N(*Cx3cr1−/−* affected F3) = 16, N(*Cx3cr1−/−* unaffected F3) = 71, N(new Ccl2−/−/*Cx3cr1−/−* affected F3) = 19, N(new Ccl2−/−/*Cx3cr1−/−* unaffected F3) = 32.

To exclude a potential genetic background effect that might contribute to some of the observed variability between different chemokine knockout lines and to understand whether and how combined defects in both chemokine pathways in *CCDKO* mice contribute to retinal degeneration, we generated *Ccl2^−/−^*, *Cx3cr1^−/−^* and *Ccl2^−/−^/Cx3cr1^−/−^* mouse lines by backcrossing the original parental line with *C57BL/6* mice (figure 6A). We set up three independent F1 breeding pairs from offspring of our parental crosses and obtain 85 F2 offspring. Genotyping of all 85 offspring for the *Ccl2* and *Cx3cr1* alleles resulted in all expected genotype combinations in a ratio consistent with two independent Mendelian traits (table 2).

**Table 2:** Genotype distribution for both chemokine knockout alleles in the F2 generation obtained from three independent F1 breeding pairs during the backcross of the original *CCDKO* mice with *C57Bl/6* mice.

Genotype for *Ccl2* and *Cx3cr1*	*Ccl2^+/+^*	*Ccl2^+/−^*	*Ccl2^−/−^*	Sum
***Cx3cr1^+/+^***	13	20	**11**	**44**
***Cx3cr1^+/−^***	5	18	5	**28**
***Cx3cr1^−/−^***	**5**	6	**2**	**13**
**Sum**	**23**	**44**	**18**	**85**

The number of animals with respective genotype combinations for both chemokine loci is shown in the field at crossing of columns (*Ccl2* genotype) and rows (*Cx3cr1* genotype). Founder animals for newly re-derived *Ccl2*, *Cx3cr1* single and *Ccl2/Cx3cr1* double knockout lines are underlined.

By phenotyping a subset of the obtained chemokine knockout mice (underlined in table 2) using autofluorescent AF-SLO imaging, we observed that the typical autofluorescent fundus lesions in these mice did not co-segregate with any of the chemokine genotypes, but that the inheritance pattern for this phenotype was consistent with an independent third autosomal recessive locus in these mice (table 3).

**Table 3:** The distribution of the inferior autofluorescent fundus lesions in phenotyped animals of the F2 generation suggests that this phenotype segregates independently from either of the chemokine loci in an autosomal recessive fashion.

Genotype	N (F2 animals phenotyped)	N (F2 animals with inferior AF lesions)	N (F2 animals without inferior AF lesions)
***Ccl2^−/−^/Cx3cr1^+/+^***	6	2	4
***Ccl2^+/+^/Cx3cr1^−/−^***	5	2	3
***Ccl2^−/−^/Cx3cr1^−/−^***	2	0	2
***All F2 animals***	**13 (100%)**	**4 (31%)**	**9 (69%)**

Subsequent breeding of F2 mice using a combination of autofluorescent fundus imaging at 8 weeks of age with genotyping for both chemokine loci of all offspring (F3 generation) allowed us to establish 6 independent chemokine knockout mouse lines. For each chemokine genotype including *Ccl2* single knockout mice (***Ccl2^−/−^/Cx3cr1^+/+^***), *Cx3cr1* single knockout mice ***Ccl2^+/+^/Cx3cr1^−/−^***), and the re-derived *Ccl2/Cx3cr1* double knockout mice (***Ccl2^−/−^/Cx3cr1^−/−^***) we established affected and an unaffected mouse lines that either exhibit the typical autofluorescent fundus lesions at the age of 8 weeks or not (figure 6B). To ensure, that we have not missed a late-onset phenotype developing in either of the newly established chemokine lines, we also imaged animals of all these new lines at the age of 6–9 months and did not observe any inferior autofluorescent lesion in all three “unaffected” lines, while we always observed typical autofluorescent changes in the inferior part of the retina of animals from the “affected” lines (figure 6C). This strongly suggested that the early onset, inferior retinal degeneration characterised by the appearance of the typical disciform autofluorescent lesion in the inferior retina is caused by a third genetic locus, that leads to a typical retinal degeneration phenotype and indicates that the original *CCDKO* double knockout mouse line for *Ccl2* and *Cx3cr1* actually contains a third autosomal recessive retinal degeneration locus. By genomic sequencing of affected and unaffected animals from all 6 lines, we found that the naturally occurring RD8 mutation, a single base pair deletion in exon 9 of the *Crb1* gene, co-segregates 100% with the observed early-onset inferior retinal degeneration in the original *CCDKO* mouse line as well as in all three re-derived “affected” *Ccl2^−/−^*, *Cx3cr1^−/−^* and *Ccl2^−/−^/Cx3cr1^−/−^* “single” and “double” chemokine knockout mouse lines.

### The genetic background as well as *Ccl2* and *Cx3cr1* chemokine ablation differentially modulates the retinal degeneration caused by the autosomal recessive homozygous *Crb1^RD8/RD8^* mutation

The genetic proof of a third autosomal recessive locus by breeding, genotyping and phenotyping of the lines as well as the presence of *Crb1*-RD8 mutation in all affected lines, allowed us to subsequently use these lines to assess the differential modulatory effect of the genetic background and the two chemokine loci, *Ccl2* and *Cx3cr1*, on the phenotype of the RD8 retinal degeneration in offspring from the F2 generation. As a quantitative measure for the retinal degeneration phenotype, we decided to use the number of autofluorescent fundus lesions in AF-SLO fundus images at the age of 8 weeks (figure 6D). We have shown above for the original *CCDKO* mice (*Ccl2^−/−^, Cx3cr1^−/−^, Crb1^RD8/RD8^*) that these fluorescent fundus lesion are an early *in vivo* indicator for typical primary pathological events in the outer retina which led to the recruitment of microglia. Thus, assessing the number of these lesions might be a good measure for a potential modulatory effect of the chemokine knockouts on the retinal degeneration phenotype *in vivo.*

At 8 weeks of age, the original *CCDKO* line showed significantly higher number of autofluorescent fundus lesions than normal *C57Bl/6* mice and all other 6 newly established chemokine knockout mouse lines (figure 6D, One-way-ANOVA p<0.0001, with Tukey's posthoc test for multiple comparison (p<0.05)). These include all the lines that carry a homozygous RD8 mutation in combination with either of the single knockouts of *Ccl2* or *Cx3cr1* as well as the newly established “affected” chemokine double knockout mouse (*Ccl2* ^−/−^/Cx3cr1^−/−^/*Crb1^RD8/RD8^*), which has the same genotype as the original *CCDKO* line. This clearly indicates that the retinal degeneration caused by a homozygous *Crb1^RD8/RD8^* mutation in these mouse lines is attenuated by an increasing *C57Bl/6* genetic background.

Furthermore, it became evident that the homozygous *Crb1^RD8/RD8^* mutation indeed causes the early onset retinal phenotype since all mouse lines that carry the *Crb1^RD8/RD8^* mutation (figure 6D, “affected”) also show a significantly higher number of autofluorescent lesions in their fundus images not only compared to *C57Bl/6* control mice, but also in comparison to all the corresponding single or double chemokine knockout mice (*Ccl2^−/−^*, *Cx3cr1^−/−^*, *Ccl2^−/−^/Cx3cr1^−/−^*) that do not carry the *Crb1^RD8/RD8^* mutation (figure 6D “unaffected”). Inversely, this shows that neither the single nor the double knockout of the chemokines *Ccl2* and/or *Cx3cr1* does lead to an early onset inferior retinal degeneration since all unaffected lines show a similar very low number of any autofluorescent lesions in the fundus as wildtype mice (figure 6D).

By comparing the affected mouse lines with each other we observed that the re-derived *Ccl2^−/−^/Cx3cr1^−/−^/Crb1^RD8/RD8^* mice (new Ccl2−/−/Cx3cr1−/− affected F3) and *Cx3cr1^−/−^/Crb1^RD8/RD8^* mice (Cx3cr1−/− (affected) F3) both showed similar numbers of autofluorescent fundus lesions, which was for both lines significantly higher that the number of autofluorescent fundus lesions in *Ccl2^−/−^/Crb1^RD8/RD8^* mice (Ccl2−/− (affected) F3.

This suggests that absence of Cx3cr1 in either the single (*Cx3cr1^−/−^/Crb1^RD8/RD8^*) or the double chemokine knockout (*Ccl2^−/−^/Cx3cr1^−/−^/Crb1^RD8/RD8^*) leads to a more pronounced degenerative phenotype of the RD8 mutation than the RD8 mutation in combination with the knockout of *Ccl2* (*Ccl2^−/−^/Crb1^RD8/RD8^*) alone. Furthermore this also shows, that there is no significant additive affect of knocking *Ccl2* out in addition to Cx3cr1, since the double chemokine knockout of *Ccl2* and *Cx3cr1* (*Ccl2^−/−^/Cx3cr1^−/−^/Crb1^RD8/RD8^*) is not significantly increased compared to the single knockout of *Cx3cr1* (*Cx3cr1^−/−^/Crb1^RD8/RD8^*) (figure 6D).

**Figure 7:**
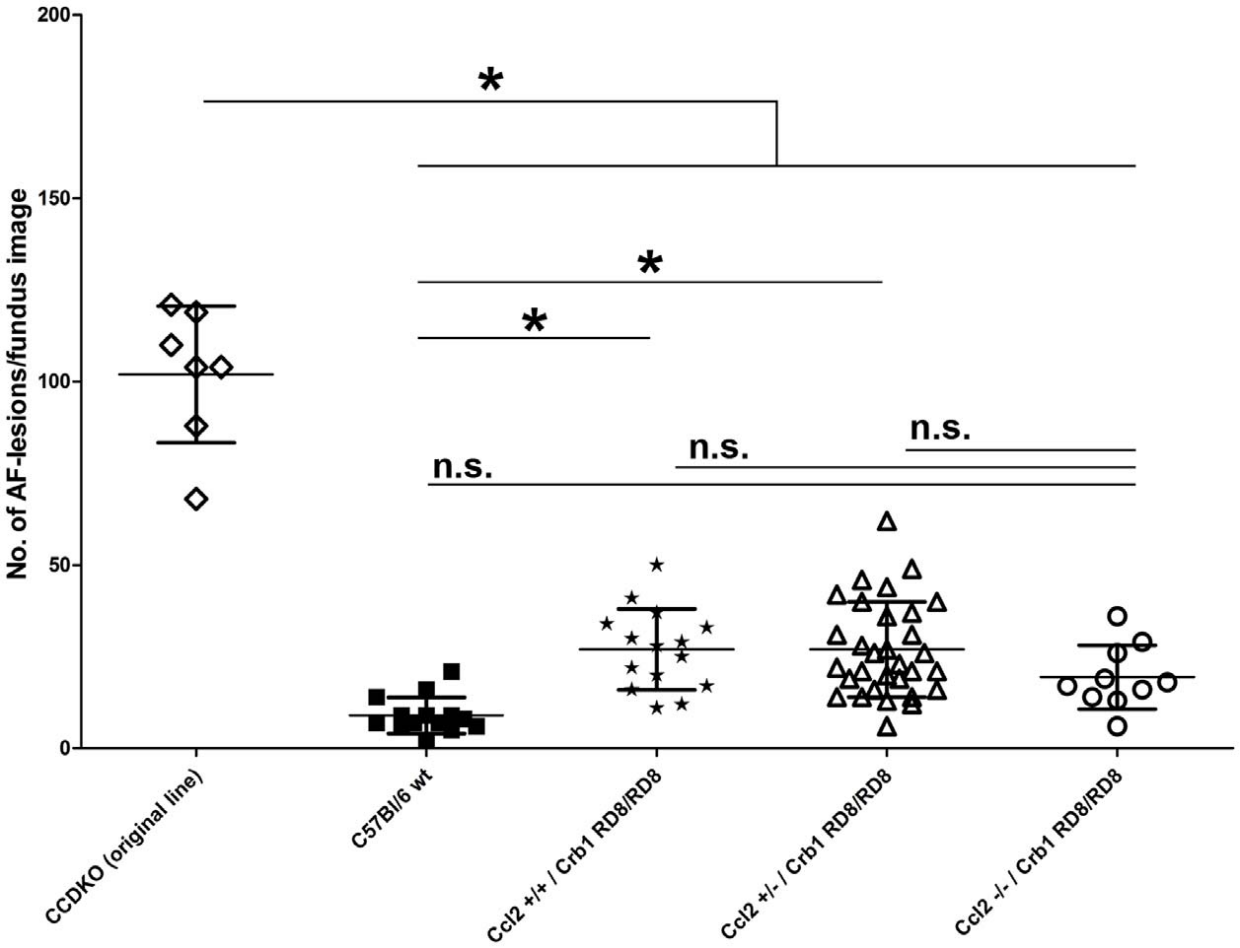
Modulatory effect of the *Ccl2* locus genotype (*Ccl2^+/+^, Ccl2^+/−^, Ccl2^−/−^*) on the appearance of autofluorescent fundus lesions in *Crb1^RD8/RD8^ mice.* Quantification of number of autofluorescent lesions/fundus image and littermate (n = ) according to the *Ccl2* genotype and in comparison to the parental strains (*CCDKO* and *C57Bl/6* mice). These findings indicate that the *Ccl2* locus does not significantly modulate the severity of RD8 induced retinal degeneration although we can not exclude a mild influence due to the intermediate position of the *Ccl2^−/−^/Crb1^RD8/RD8^* group. *: indicates significant difference between the indicated groups (One-way-ANOVA p<0.0001, Tukey's posthoc test for multiple comparison (p<0.05)). n.s.: not significant. N (*CCDKO* original line P) = 7, N(*C57Bl/6 wt* P) = 14, N(*Ccl2^+/+^/Crb1^RD8/RD8^*) = 15, N(*Ccl2^+/−^/Crb1^RD8/RD8^*) = 31, N(*Ccl2^−/−^/Crb1^RD8/RD8^*) = 10.

To clarify, whether absence of *Ccl2* signalling alone might also have a modulatory effect on the RD8 retinal degeneration, we evaluated littermates from the backcrosses that all carried the *Crb1^RD8/RD8^* mutation but were either homozygous wildtype (*Ccl2^+/+^*/*Crb1^RD8/RD8^*), heterozygous (*Ccl2^+/−^*/*Crb1^RD8/RD8^*) or homozygous knockout (*Ccl2^−/−^*/*Crb1^RD8/RD8^*) for the *Ccl2* locus (figure 7). These animals were obtained from two specific breeding pairs whose parental mice were heterozygous for *Ccl2*, wildtype for *Cx3cr1* and homozygous for the RD8 mutation (*Ccl2^+/−^/Cx3cfr1^+/+^/Crb1^RD8/RD8^*). All offspring with any of the *Ccl2* genotype combinations showed a significantly smaller number of autofluorescent lesions in the fundus compared to the original *CCDKO* mice (figure 7). This reflects the combinatory effect of the additional *C57Bl/6* genetic background and of the homozygous *Cx3cr1^−/−^* knockout allele on the retinal degeneration caused by the homozygous RD8 alleles (figure 7). Compared with independent *C57Bl/6* wildtype mice (figure 7, *C57Bl/6* wt), littermates from the backcross that carried the homozygous RD8 mutation, but were wildtype (figure 7, *Ccl2^+/+^/Crb1^RD8/RD8^*) or heterozygous (figure 7, *Ccl2^+/−^/Crb1^RD8/RD8^*) for *Ccl2* showed a significantly higher number of lesions in the fundus, while Ccl2 deficient littermates (figure 7, *Ccl2^−/−^/Crb1^RD8/RD8^*) show no significant difference compared with all three groups at 8 weeks of age. Therefore this data suggests that *Ccl2* does not have a significant effect on the manifestation of the early onset retinal degeneration in mice that carry a homozygous RD8 mutation, although we cannot exclude a mildly protective effect of *Ccl2* deficiency.

## Discussion

In this study, we aimed to identify primary and secondary pathological events during the previously described early onset retinal degeneration in *Ccl2/Cx3cr1* double knockout (*CCDKO*) mouse line and to understand whether and how these two chemokine pathways contribute to the retinal degeneration [41]. We show that the early onset retinal degeneration in the original *CCDKO* mouse line [41] is focally localised in the inferior retina and that the primary pathological event is an early drop out of nuclei from the outer nuclear layer. The RPE and the retinal vasculature are affected secondarily at later stages during disease progression. These findings are consistent with a very recent report from another group which also identified primary lesions near the outer limiting membrane in the same *CCDKO* line and suggested an involvement of Müller cells in disease progression [50]. We now provide evidence that the early onset retinal degeneration, observed in these *CCDKO* knockout mouse lines as well as in all re-derived “affected” *Ccl2* and *Cx3cr1* and *Ccl2/Cx3cr1* double knockout mice, is caused by a third independent autosomal recessive locus. Re-derived *Ccl2* or *Cx3cr1* single knockout lines and re-established *Ccl2/Cx3cr1* double knockout mice which do not carry the third locus exhibited no signs of an early onset retinal phenotype. This indicates that neither *Ccl2* nor *Cx3cr1* deficiency alone nor the combined double knockout of both chemokines leads to a pronounced early onset retinal degeneration. In addition we showed by genomic sequencing that the naturally occurring homozygous RD8 mutation co-segregates with the early onset retinal phenotype in all affected chemokine lines. This naturally occurring RD8 (*Crb1^RD8/RD8^*) mutation is a single base pair deletion of a cytosine in exon 9 of the *Crb1* gene that causes a frame shift and premature stop codon in the Crb1 protein which lead to a truncated transmembrane and cytoplasmic domain of Crb1 [51]. Crb1 is a key regulator of epithelial cell polarity and contributes to the assembly of the zonula adherens, a belt like adherent junction that separates apical and basolateral membranes [51]. In the retina, Crb1 resides in Müller cells and is crucially involved in the formation of the outer limiting membrane at the posterior site of the outer nuclear layer of the retina. Lack of Crb1 protein in *Crb1^RD8/RD8^* mice leads to very similar phenotypic features as we have observed in *CCDKO* mice. These include the early onset inferior fundus lesions, a discontinuous outer limiting membrane, an early dropout of nuclei from the outer nuclear layer as well as the subsequent focal age-related retinal dysplasia which is secondarily associated with vascular lesions in the outer retina [51]–[53]. Furthermore, also our observation that the initiation of the inferior retinal degeneration in *CCDKO* mice is independent from light is consistent with previous data for *Crb1^−/−^* knockout mice. These mice, when raised in complete darkness for 6 months also show a similar degree of retinal degeneration as *Crb1^−/−^* mice raised under normal lighting conditions (100 lux) [53]. However, increased white fluorescent light levels of 3000 lux for 72 hours promote the retinal degeneration in *Crb1^−/−^* mice [52] suggesting that the ambient light conditions, we used in this study for *CCDKO* mice are about 100fold lower than light levels necessary to modulate the phenotype of this type of retinal degeneration. These data suggest, that the inferior location of the retinal degeneration in *Crb1* mutant mice (*Crb1^RD8/RD8^*, *Crb1^−/−^* and *CDDKO* mice) is not due to the influence of light, but due to an unknown factor that restricts the photoreceptor degeneration to the inferior retina. These pronounced similarities in the phenotype as well as the co-segregation of the *Crb1^RD8/RD8^* mutation with the early onset retinal degeneration in the different re-derived chemokine knockout mice indicate that the retinal degeneration observed in original *CCDKO* mice is the consequence of the *Crb1^RD8/RD8^* mutation and not due to the combined double knockout of *Ccl2* and *Cx3cr1* as previously reported [41].

### Late stage retinal degeneration in *Ccl2^−/−^/Cx3cr1^−/−^/Crb1^RD8/RD8^* mice leads to the development of retinal vascular lesions similar to retinal telangiectasia

The identification of the *Crb1^RD8/RD8^* mutation as the underlying cause for the early onset retinal degeneration in *CCDKO* mice enables us to better interpret the observed secondary degenerative events in original *CCDKO* mice. The two major secondary events observed in the *Ccl2^−/−^/Cx3cr1^−/−^/Crb1^RD8/RD8^* mice with age include RPE changes and closely associated vascular lesions in the area of retinal degeneration. The vascular lesions contained tortuous and dilated vessels in the area of retinal degeneration and showed an increasing penetrance of late phase hyperfluorescence in fluorescein angiography which indicates a low grade vascular inflammation and a low grade late vascular leakiness of retinal vessels within the lesions. Using serial block-face scanning electron microscopy (SB-SEM) and 3D reconstruction, we demonstrated that the retinal vessels within the degenerate area break through the RPE layer and come in close contact with Bruch's membrane, but do not penetrate it. This abnormal location of retinal vessels seems to induce a secondary response of RPE cells which migrate along those vessels into the retina and ensheath them within the RPE/vascular lesion. However, the fact that not all aged *CCDKO* mice showed the occurrence of the late phase hyperfluorescence in the fluorescein angiography suggests that the manifestation of this vascular phenotype is variable and thus likely not a primary consequence of the RD8 mutation, but rather a secondary process dependent on the individual severity of the progression of the retinal degeneration. Based on the clinical observations (late phase FFA hyperfluorescence, tortuous and dilated vessels within the degenerate areas of the retina) as well as on the basis of our ultrastructural observations, that vessels of retinal origin grow underneath the RPE but do not penetrate Bruch's membrane, we propose that the vessels within the RPE/vascular lesions in the *Ccl2^−/−^/Cx3cr1^−/−^/Crb1^RD8/RD8^* mice are similar to retinal telangiectasia rather than choroidal neovascularisation, which was reported to occur in about 10–15% of the original *CCDKO* mouse line above the age of 3 months [41]. Our findings are also consistent with the recently reported stabilization of the lesion phenotype by virus mediated overexpression of sFLT1 which might attenuate the secondary low grade vascular inflammation and leakiness during disease progression and thus might lead to the reported beneficial outcome for *CCDKO* mice [43].

Since the late stage secondary changes in *CCDKO* mice are also very similar to those in aged *Crb1^RD8/RD8^* mice [53], we suggest that *Crb1^RD8/RD8^* mice might show similar vascular lesions as described here and do not develop CNV. Our data supports the view that vascular lesions in patients with *Crb1* mutations, that show Coat's-like exudates in the inferior region of the retina, are likely to be more similar to retinal telangiectasia with similar vascular features as observed in this study, than choroidal neovascularisation [54]. Future ultrastructural studies using serial block-face scanning electron microscopy- (SB_SEM) and 3D reconstruction may help to elucidate the nature of vascular lesions in both *Crb1^RD8/RD8^* mice as well as in patients with *Crb1* mutations. A similar process in which retinal vessels are located adjacent to Bruch's membrane and are covered by RPE cells has been described in late stage retinal degeneration in rhodopsin knockout mice [55] suggesting that the development of this type of RPE/vascular lesions may be not only specific to retinal degeneration caused by mutations in *Crb1*, but might be more common during late stages of other severe retinal degenerations.

### Genetic background modulates the retinal degeneration caused by RD8 mutation in *CCDKO* mice

Our data demonstrates that genetic backcrossing of the *CCDKO* mice to a *C57Bl/6* genetic background attenuates the observed retinal degeneration in *CCDKO* mice. The data suggest that the genetic background of *C57Bl/6* is a strong modulator of the manifestation of retinal degeneration in all affected chemokine knockout mice carrying the *Crb1^RD8/RD8^* mutation and indicates that other genetic factors influence the observed phenotype. The presence of other genetic modifiers is also supported by the high degree of phenotypic variability observed within each affected mouse lines. Our findings are consistent with the original characterisation of *Crb1^RD8/RD8^* mice, which demonstrated that the retinal degeneration is strongly modulated by backcrosses into *C57Bl/6, CAST/EiJ* or C*3HfB6/Ga* mice. Interestingly, only the discontinuous outer limiting membrane phenotype in *Crb1^RD8/RD8^* mice remained preserved after backcrossing [51] supporting that this may be the primary consequence of the *Crb1^RD8/RD8^* mutation in *Ccl2^−/−^/Cx3cr1^−/−^/Crb1^RD8/RD8^* mice in this study as well.

### *Ccl2 and Cx3cr1* chemokine signalling differentially modulate retinal degeneration in *Crb1^RD8/RD8^* mice

Our data demonstrates that the genetic inactivation of either *Ccl2 and/or Cx3cr1* chemokine signalling differentially modulates the severity of the retinal degeneration caused by the *Crb1^RD8/RD8^* mutation at 8 weeks of age. This is supported by the observation that all *Cx3cr1* deficient lines that carry the *Crb1^RD8/RD8^* mutation, including the *Ccl2^−/−^/Cx3cr1^−/−^/Crb1^RD8/RD8^* and *Cx3cr1^−/−^/Crb1^RD8/RD8^* mice, exhibit a more severe retinal degeneration compared to the *Ccl2^−/−^/Crb1^RD8/RD8^* line of the same backcross generation and age. In contrast, *Ccl2* deficiency (*Ccl2^−/−^/Crb1^RD8/RD8^*) alone does not significantly influence the retinal degeneration caused by the *Crb1^RD8/RD8^* mutation, although we can not exclude a slight protective effect of it based on the trend for a reduced number of autofluorescent lesions in *Ccl2* deficient mice (*Ccl2^−/−^/Crb1^RD8/RD8^*) compared to heterozygous (*Ccl2^+/−^/Crb1^RD8/RD8^*) or wildtype (*Ccl2^+/+^/Crb1^RD8/RD8^*) littermates. *Ccl2* deficiency also does not significantly add to the exacerbated retinal degeneration seen in *Cx3cr1^−/−^/Crb1^RD8/RD8^* mice suggesting that the effects of *Ccl2* and *Cx3cr1* signalling defects do not act synergistically on the manifestation of the retinal degeneration in *Crb1^RD8/RD8^* mice. A modulatory influence of either of the two chemokine pathways and in particular of *Cx3cr1* signalling on retinal degenerations, is further supported by the observation that Iba1^+^ microglia cells, which express *Cx3cr1* in the retina [1], are recruited to the primary site of pathology and interact with the degenerating cells in the outer retina.

Our findings support the hypothesis that in original *CCDKO* line and in other affected chemokine lines in this study, the defective formation of the outer limiting membrane due to mutations in *Crb1* acts as an endogenous trigger for the local upregulation of inflammatory mediators. This hypothesis is consistent with the increased expression of *Cx3cl1* in retinal and choroidal vascular endothelial cells, Müller cells, RPE as well as photoreceptors after inflammatory processes [56] as well as with the observed upregulation of *Ccl2* in Müller cells within affected retinal areas after focal light injury and after retinal detachment [57], [58]. Both, *Ccl2* as well as *Cx3cl1* provide chemotactic cues for the local recruitment of microglia and systemic monocytes to the primary focal lesion site [12], [23], [59] and are part of a local chemokine signalling network between photoreceptor cells and Müller cells that controls the local recruitment and activation of microglia and systemic macrophages in the retina. Dependent on which and how severely each of the chemokine pathways are affected by endogenous genetic variants within this cellular signalling network, the responses of recruited microglia or monocytes will differ and can either exacerbate retinal degeneration, as observed for *Cx3cr1* deficient mice in combination with the *Crb1^RD8/RD8^* mutation, or may act mildly protective as eventually indicated by the trend towards a slight reduction in lesion size in *Ccl2* deficient mice which also carry the *Crb1^RD8/RD8^* mutation.

Further support for this idea comes from convincing evidence for a microglia-mediated neurotoxicity in *Cx3cr1* knockout mice in the brain, where an increased neurotoxicity of brain microglia on surrounding neurons has been observed after systemic inflammation induced by LPS [28]. In the retina, *Cx3cr1* deficiency leads to a reduced dynamic behavior of retinal microglia during immune surveillance as well as during injury response [30]. It has also been found to be associated with an increased photoreceptor degeneration during ageing and after light exposure [29] supporting the hypothesis that *Cx3cr1* deficient microglia can be neurotoxic in the retina as well and have the capacity to act as modulators of retinal degeneration as suggested by our observation. Therefore, we hypothesise that the additional neurotoxic effect of *Cx3cr1* deficiency on the retinal degeneration caused by the *Crb1^RD8/RD8^* mutation might be due to an impaired migratory capability and a subsequent reduced removal of dying photoreceptors by *Cx3cr1-*deficient microglia which might result in the production of more neurotoxic inflammatory mediators including Ccl5, TNFa or IL-6, which may negatively affect photoreceptor cell survival, and have all been previously shown to be upregulated in *CCDKO* mice [28], [30], [42], [60], [61]. In contrast to the effect of *Cx3cr1* deficiency, *Ccl2* deficiency does not significantly modulate the manifestation of the *Crb1^RD8/RD8^* phenotype. This minor role of *Ccl2* on the RD8 retinal degeneration seems consistent with our previous observation that *Ccl2* deficiency does not lead to a significant associated age-related retinal degeneration despite an accumulation of subretinal, dysfunctional macrophages [40] and may be explained by a minor role of *Ccl2* in this process or by a compensatory effect of other chemokine ligands that can bind to and activate CCR2 [19]. However, a slightly but not significantly reduced number of autofluorescent lesions in *Ccl2^−/−^/Crb1^RD8/RD8^* mice may suggest a mild protective effect of *Ccl2* deficiency, which would be in line with *Ccl2's* role for the transendothelial migration of pro-inflammatory monocytes across the blood brain barrier [25]. This process could be similar to the protective effect of *Ccl2* and *Ccr2* deficiencies for the formation of atherosclerosis that lead to a reduction of the formation of foam cells due to a reduced recruitment of systemic myeloid cells to the site of the lesion in the periphery [62].

Taken together, our study provides evidence for a differential modulatory role of *Ccl2* and *Cx3cr1* chemokine signalling for the inherited retinal degeneration caused by the *Crb1^RD8/RD8^* mutation and suggests that these two chemokine pathways not only modulate age-related degenerative processes or acute inflammation in the retina, but also contribute to the pathology of inherited retinal degenerations. Therefore it seems likely that these two chemokine signalling pathways and maybe the innate immune status of myeloid cells might act as a modifier for *CRB1* mutations in humans and thus contribute to the high phenotypic variability observed in patients with different *CRB1* mutations that either lead to Lebers congenital amaurosis (LCA), early onset childhood retinal dystrophy or juvenile onset retinitis pigmentosa [54]. Recent reports of increased *CCL2* levels and reduced expression of *Cx3cr1* on non-classical monocytes in aged probands as well as further increased levels of *CCL2* in aqueous humor samples from patients with exudative AMD [35], [36] and in patients with advanced proliferative diabetic retinopathy [63] suggest that these two chemokine pathways reveal an age-related shift from *CX3CL1-CX3CR1* signalling to increased *CCL2-CCR2* signalling systemically with age which might contribute to an increased susceptibility to retinal degeneration with age and during disease progression of multi-factorial age-related retinal diseases including AMD and diabetes. Understanding further the role and interaction of chemokine signalling pathways for the control of monocytes and microglia cell in degenerative processes of the retina might help to develop more targeted therapeutic approaches to modulate the progression of inherited and other age-related, multi-factorial retinal degenerations.

## Supporting Information

Movie S1 ***C57Bl/6*** **.** Animation of 3D-reconstructions (IMARIS) of confocaol Z-stack images taken from the inferior area of a retinal flat mount of a *C57Bl/6* mouse at 2 months of age. The volume of the z-stack (x = 183.3 µm, y = 183.3 µm, z = 76 µm) comprises the deep retinal vascular plexus, the outer nuclear layer and part of the inner segments. The deep retinal vascular plexus was labeled with Tritc-lectinB4 and is represented in red, while retinal microglia labeled with Iba1 are shown in green. At the top of the z-stack microglia located at the level of the deep retinal vascular plexus show a normal ramified morphology. These cells are not present in the outer retina. The red and green Imaris surfaces in the area of the inner segments at the bottom of the z-stack represent autofluorescent signals from photoreceptors.
(AVI)

Movie S2 **CCDKO.** Animation of 3D-reconstructions (IMARIS) of confocaol Z-stack images taken from the inferior area of a retinal flat mount of a *CCDKO* mouse at 2 months of age. The volume of the z-stack (x = 183.3 µm, y = 183.3 µm, z = 76 µm) comprises the deep retinal vascular plexus, the outer nuclear layer and part of the inner segments. The deep retinal vascular plexus was labeled with Tritc-lectinB4 and is represented in red, while retinal microglia labeled with Iba1 are shown in green. At the top of the z-stack microglia located at the level of the deep retinal vascular plexus show thicker cell bodies compared to wildtype. In addition microglia are also observed in the outer retina in distinct columns. These microglia columns are observed in areas were the inner segment autofluorescence (at the bottom of the z-stack) is absent indicating altered photoreceptor positions or drop out.
(AVI)
